# Projected loss of soil organic carbon in temperate agricultural soils in the 21^st^ century: effects of climate change and carbon input trends

**DOI:** 10.1038/srep32525

**Published:** 2016-09-02

**Authors:** Martin Wiesmeier, Christopher Poeplau, Carlos A. Sierra, Harald Maier, Cathleen Frühauf, Rico Hübner, Anna Kühnel, Peter Spörlein, Uwe Geuß, Edzard Hangen, Bernd Schilling, Margit von Lützow, Ingrid Kögel-Knabner

**Affiliations:** 1 Chair of Soil Science, TUM School of Life Sciences Weihenstephan, Technical University of Munich, 85354 Freising, Germany; 2 Thuenen Institute for Agricultural Climate Research, Bundesallee 50, 38106 Braunschweig, Germany; 3 Max Planck Institute for Biogeochemistry, Hans-Knöll-Str. 10, 07745 Jena, Germany; 4 Deutscher Wetterdienst, Abteilung Agrarmeteorologie, Niederlassung Weihenstephan, Alte Akademie 16, 85350 Freising-Weihenstephan, Germany; 5 Deutscher Wetterdienst, Abteilung Agrarmeteorologie, ZAMF, Bundesallee 50, 38116 Braunschweig, Germany; 6 Chair for Strategic Landscape Planning and Management, TUM School of Life Sciences Weihenstephan, Technical University of Munich, 85354 Freising, Germany; 7 Bavarian Environment Agency, 95030 Hof, Germany; 8 Institute for Advanced Study, Technical University of Munich, 85748 Garching, Germany

## Abstract

Climate change and stagnating crop yields may cause a decline of SOC stocks in agricultural soils leading to considerable CO_2_ emissions and reduced agricultural productivity. Regional model-based SOC projections are needed to evaluate these potential risks. In this study, we simulated the future SOC development in cropland and grassland soils of Bavaria in the 21^st^ century. Soils from 51 study sites representing the most important soil classes of Central Europe were fractionated and derived SOC pools were used to initialize the RothC soil carbon model. For each site, long-term C inputs were determined using the C allocation method. Model runs were performed for three different C input scenarios as a realistic range of projected yield development. Our modelling approach revealed substantial SOC decreases of 11–16% under an expected mean temperature increase of 3.3 °C assuming unchanged C inputs. For the scenario of 20% reduced C inputs, agricultural SOC stocks are projected to decline by 19–24%. Remarkably, even the optimistic scenario of 20% increased C inputs led to SOC decreases of 3–8%. Projected SOC changes largely differed among investigated soil classes. Our results indicated that C inputs have to increase by 29% to maintain present SOC stocks in agricultural soils.

Soil organic carbon (SOC) represents the largest carbon pool in terrestrial ecosystems and is a key factor that controls important soil functions, e.g. the productivity of agricultural soils^1^. The maintenance of SOC stocks in croplands and grasslands of the world is thus of upmost importance for ensuring global food security and the prevention of substantial CO_2_ emissions. Under long-term constant management and environmental conditions, agricultural SOC stocks are in a dynamic equilibrium between C inputs, mainly in form of crop residues and organic fertilizers, and a loss of C due to decomposition of soil organic matter (SOM). However, as the decomposition of SOM is strongly controlled by temperature and soil moisture, climate change imposes the risk of SOC losses^2^,^3^,^4^,^5^,^6^. Both a the global and regional scale decreasing SOC stocks were observed along temperature gradients from colder to warmer regions^7^,^8^. This suggests that SOM decomposition rates change faster as a function of temperature than does net primary production (NPP). Accordingly, rising temperatures in the course of climate change are discussed to cause significant declines of SOC. In particular, agricultural soils could be affected, as observed stagnation of crop yields and associated stagnation of C inputs in the last decades may aggravate climate change-induced SOC losses^9^,^10^. First indications for declining SOC stocks in agricultural soils were already found, but due to changes of land use and the agricultural management, a relationship with climate change could not be confirmed to date^11^,^12^,^13^,^14^,^15^,^16^,^17^.

The potential risk of SOC losses induced by climate change calls for a projection of agricultural SOC stocks under future climate change scenarios on the basis of SOC models. A frequently applied model is the Rothamsted Carbon Model (RothC) which was specifically designed to simulate SOC dynamics in temperate cropland and grassland soils^18^. Several modelling approaches were conducted to simulate regional or global development of agricultural SOC stocks^19^,^20^,^21^,^22^,^23^,^24^,^25^. However, these SOC projections were often based on simple estimations of important input variables or legacy data with a relatively low spatial resolution. In particular, rough estimations for the C input were used, which is one of the most decisive parameters in SOC projections. As the determination of the total C input, particularly the belowground components such as roots and rhizodeposition, is difficult and elaborate, values are generally estimated using an inverse application of the RothC model or simply gross values from the literature^18^,^20^,^26^,^27^. However, large discrepancies were observed between measured and estimated C input values and thus an independent, more reliable approach is needed^28^. Future projections of the development of C inputs in agricultural soils are even more challenging due to overlapping implications of crop and land use management, future trend of crop breeding and technology and climate change. In several SOC modelling studies it was assumed that the development of agricultural C inputs are simply related to NPP development, which can be simulated by vegetation models^19^,^20^,^21^,^22^,^25^,^29^,^30^,^31^. However, such an approach neglects the fact that the C input in agricultural soils is controlled by various factors including management practices. Moreover, reliable SOC projections using the RothC model require a reasonable initialization of the model. In terms of model initialization, SOM pools derived from soil fractionation is a laborious, but probably the most suitable approach as this method reflects the actual state of SOC pools^27^,^32^,^33^,^34^.

In this study, we simulated the future development of SOC stocks of cropland and grassland soils in Bavaria from 2000 to 2095 under a moderate climate scenario of the IPCC (A1B) using a large number of investigated sites (Fig. 1). In total, 21 cropland and 30 grassland sites representing most important soil classes of Central Europe were sampled and SOC pools were fractionated following the approach of Zimmermann *et al*.^34^, who were able to empirically link SOC fractions to RothC pools. In a preliminary study, C inputs of major crops and grassland in Bavaria were determined with a high spatial and temporal resolution^35^, which enabled a calculation of site-specific C input values. The RothC model was then used to simulate SOC development under three different C input scenarios, which covered the range of expected crop yield development in Bavaria.

## Results

### Climate conditions at study sites and projected changes

The ensemble approach on the basis of 19 climate models revealed reliable spatially differentiated projections of air temperature, precipitation and evapotranspiration for all studied sites (Table 1, Fig. 2). In general, cropland sites exhibited average long-term values (1971 to 1999) for mean annual temperature of 8.3 to 8.9 °C, for mean annual precipitation of 831 to 873 mm and for mean annual evapotranspiration of 707 to 760 mm. Between 2000 and 2095 climate projections under the A1B climate scenario revealed increases in air temperature by 3.1 to 3.4 °C, in precipitation by 35 to 82 mm and in evapotranspiration by 80 to 123 mm. Due to their main distribution in pre-alpine regions, grassland sites had slightly lower mean annual temperatures of 7.6 to 8.7 °C, considerably higher values of mean annual precipitation of 846 to 1331 mm and slightly lower mean annual evapotranspiration of 663 to 739 mm. The climate projections indicated for grassland sites a temperature increase of 3.2 to 3.4 °C, a variable change of precipitation of −36 to 95 mm and an increase of evapotranspiration of 77 to 143 mm.

### Basic soil properties, SOC fractions and C input

Basic soil properties and SOC fractions for each soil unit under cropland and grassland are given in Table 2. In general, cropland soils revealed higher thicknesses of the A horizon (29 ± 6 cm) compared to grassland soils (23 ± 6 cm). Clay contents ranged between 21 and 52% in soil units under cropland and from 19 to 45% under grassland. The contribution of OC in different soil fractions to bulk SOC stocks differed strongly among the investigated soil/land use units. Generally, DOC contributed with only 2 to 7% to total SOC stocks of both cropland and grassland topsoils. Considerable differences between soil units were found for the POM fraction that contained 5 to 21% of total SOC stocks in soils under cropland and 3 to 16% under grassland. The highest POM-OC amounts were found for Cambisols from Tertiary material (C1) as well as from sandstone (C6, C7). For the S+A fraction distinct differences were found between cropland and grassland soil units. Under cropland, S+A contained only 2 to 10% of bulk SOC with the exception of groundwater-affected soils (G) that stored 16%. In contrast, grassland soil units revealed much higher OC amounts in the S+A fraction (16 to 34% of bulk SOC) with highest amounts in groundwater-affected soils (G, 57%). The major part of SOC was found in the s+c fraction that contained 50 to 83% of total SOC stocks independent from land use (only groundwater-affected soils under grassland revealed a considerably lower contribution of 30%). The inert rSOC fraction contained OC amounts of 3% to 10% of total SOC stocks without distinct differences between cropland and grassland soil units. In total, soil units under cropland revealed significantly (P < 0.05) lower SOC stocks in A horizons (59 ± 21 t ha^−1^) than grassland soils (72 ± 24 t ha^−1^). The calculated mean C input values derived from the period from 1995 to 1999 showed no significant differences among soil units under cropland and grassland. The C inputs ranged between 3.4 and 4.1 t ha^−1^ in cropland soils and from 4.5 to 5.3 t ha^−1^ in grassland soils. Over a 16 year period from 1995 to 2010, C inputs in cropland showed no significant trend but remained on a constant level (Fig. 3). In grasslands soils, C inputs revealed a slight increase of 8% (based on a linear trend with an R^2^ value of 0.23).

### Projected development of SOC stocks under different C input scenarios

The projected development of SOC stocks of cropland and grassland soils under the reference scenario and different C input scenarios are shown in Figs 4 and 5. In cropland soils, the reference climate scenario (constant average climate conditions 1970–1999) showed an increase of mean SOC stocks from 58.5 ± 20.8 t ha^−1^ in 2000 to 66.3 ± 13.9 t ha^−1^ in 2095. The projection of SOC stocks under climate change and the constant C input scenario C0 indicated a slight increase of SOC stocks until 2020 up to 61.7 ± 18.1 t ha^−1^, followed by a slight decline down to 57.2 ± 11.8 t ha^−1^ in 2095. Under climate change and C input scenario C20−, a noticeable decline of SOC stocks to 52.4 ± 11.2 t ha^−1^ in 2095 was determined after a peak of 60.9 ± 18.0 t ha^−1^ in 2020. Climate change and the C input scenario C20+ revealed a slight SOC stock increase up to 61.6 ± 12.4 t ha^−1^ in 2095.

The calculated net SOC change as the difference between the reference scenario and climate/C input scenarios showed mean losses of SOC stocks of −9.1 t ha^−1^ (−16% of initial SOC stocks) under climate change and C0, of −13.9 t ha^−1^ (−24%) under climate change and C20− and of −4.7 t ha^−1^ (−8%) under climate change and C20+ (Fig. 6). Distinct differences of SOC losses were found among the investigated soil units under cropland (Table 2). The highest relative SOC losses (compared to total SOC amount) were found for intermediate to deep soils with clay accumulation in the subsoil (L2) and Cambisols from Tertiary material (C1). For the scenarios C0, C20− and C20+, SOC stocks in these soil units decreased by −20 to −21%, −30 to −31% and −11 to −12%, respectively. For all other soil units under cropland the respective SOC decreases ranged between −12 to −17%, −19 to −26% and −2 to −9%.

For grassland sites, only a slight increase of mean SOC stocks from 71.6 ± 24.0 t ha^−1^ in 2000 to 74.9 ± 13.7 t ha^−1^ in 2095 was projected for the reference scenario (Fig. 5). The climate change and C0 scenario revealed a SOC stock decrease down to 67.4 ± 10.6 t ha^−1^ in 2095 after a peak of 73.4 ± 17.5 t ha^−1^ in 2020. SOC projections under climate change and C20− showed a considerable decline down to 61.2 ± 10.1 t ha^−1^ in 2095. A slight increase of SOC stocks up to 73.1 ± 10.9 t ha^−1^ was found for climate change and the C20+ scenario. The resulting net SOC changes accounted for −7.5 t ha^−1^ for C0 (−11% of initial SOC stocks), −13.7 t ha^−1^ for C20− (−19%) and −1.8 t ha^−1^ for C20+ (−3%) (Fig. 6). Among different soil units under grassland, distinct differences of SOC changes were detected (Table 2). In general, soil units mainly distributed in the southern part of Bavaria (groundwater-affected soils, G; soils with clay accumulation in the subsoil L1, L2; Cambisols and Luvisols from moraine material, C2) showed considerably lower SOC decreases than other investigated soil units. For C input scenarios C0 and C20−, relative SOC decreases ranged between −1 to −7% and −9 to −16%, respectively. Under scenario C20+, even SOC increases of 1 to 6% were projected for these soil units. In contrast, all other soil units under cropland revealed much higher declines of SOC of −11 to −23% for C0, −19 to −34% for C20− and −2 to −12% for C20+.

### Estimation of total SOC changes in agricultural soils of Bavaria

In order to estimate the total changes of SOC stocks for the investigated soil units on an area basis, projected mean SOC stocks from all studied sites for a soil unit were multiplied with the respective area of the soil unit (Table 3, Fig. 7). In general, cropland and grassland topsoils of Bavaria stored 122.9 Mt and 192.9 Mt SOC in 2000. In croplands, the soil units intermediate to deep Luvisols (L2) and Cambisols from Tertiary material (C1) with the largest extend in Bavaria stored 40% of total SOC stocks. Moreover, the soil units groundwater-affected soils (G), shallow soils from limestone weathering (C3) and clay-rich soils (V) contained 41% of total SOC stocks due to relatively high soil-unit-specific SOC stocks of 71.8 to 80.8 t ha^−1^. Estimations of total projected SOC changes under the scenarios C0, C20− and C20+ revealed SOC losses of 20.3 Mt, 30.9 Mt and 10.5 Mt for cropland soils, respectively. The highest absolute SOC losses were found for intermediate to deep Luvisols (L2) and Cambisols from Tertiary material (C1) due to their spatial extent and projected high decreases of SOC.

Soil units under grassland uniformly contributed to total SOC stocks. For the scenarios C0, C20− and C20+, total SOC losses of 11% of initial SOC stocks (7.6 Mt), 20% (13.7 Mt) and 3% (2.0 Mt) were estimated for grassland soils, respectively. However, for the scenario C20+ marginal increases of total SOC stocks of 0.1 to 0.3 Mt were estimated for grassland soil units in the vicinity of the Alps (groundwater-affected soils, G; soils with clay accumulation in the subsoil L1, L2; Cambisols and Luvisols from moraine material, C2). In total, the projections revealed absolute SOC losses of 27.9 Mt for the scenario C0, 44.6 Mt for C20− and 12.5 Mt for C20+ in agricultural soils of Bavaria. These SOC losses correspond to total emissions of CO_2_-equivalents of 102.3 Mt, 163.6 Mt and 45.9 Mt, respectively.

## Discussion

Reliable projections of SOC stock evolution using the RothC model require a reasonable estimation of actual SOC pool size by soil fractionation^34^. The application of proposed pedotransfer functions or equilibrium model runs as alternative methods for SOC pool size estimation may be adequate in long-term undisturbed land use systems or for long projection periods^26^,^36^, but are probably only partly suitable for intensively managed croplands and accurate projections for coming decades. Moreover, the determination of the C input on the basis of the C allocation method using crop-specific C allocation coefficients and county-specific crop yields is a promising method to estimate C inputs precisely^35^,^37^. This was confirmed by the reference scenarios (SOC projections under constant climatic conditions) that indicated only a slight overestimation of C inputs (Figs 4 and 5), assuming that SOC stocks at all investigated sites were in equilibrium. Therefore, the overall accuracy of our modelling approach can be assessed as relatively high.

Our projections of SOC stocks under future climate change and constant C inputs revealed substantial SOC losses for cropland (−16%) and grassland (−11%) in Bavaria until 2095 (Table 1, Fig. 6). The projected mean temperature increase of 3.3 °C at the study sites until 2095 and slightly increased precipitation under the A1B climate scenario will obviously lead to an increased mineralization of SOC stocks^3^,^5^. An anticipated decline of C inputs by 20% (C20−) would result in even higher decreases of SOC by 24 and 19% for cropland and grassland, respectively. Under cropland, projected SOC losses affected all investigated soil units, particularly Cambisols from Tertiary material (C1). This is mainly attributed to the fact, that these soils are characterized by relatively low clay contents (21 ± 6%). As a result, these soils contain a much lower proportion of SOC stabilized in soil aggregates and the s+c fraction and an equivalent higher contribution of labile SOC (DOC + POM) than other cropland soils (Table 2). Under grassland, slightly lower projected SOC losses compared to cropland are due to generally higher C inputs (Fig. 3) and considerably higher proportions of aggregate-protected SOC (Table 2). Among grassland soil units, noticeably lower SOC declines were projected for soils in relatively cool, pre-alpine regions with precipitation amounts >1000 mm and relatively high C input values >5 t ha^−1^. Remarkably, even under the optimistic assumption of an increase of C inputs by 20%, a decline of SOC stocks by 8 and 3% was projected for cropland and grassland, respectively (Table 1, Fig. 6). Only grassland soil units in pre-alpine regions revealed a slight SOC increase.

The finding of generally declining SOC stocks under future climate change is in line with some other SOC projection approaches for agricultural soils. Xu *et al*.^27^ modelled SOC changes in eight Irish grassland soils from 2021 to 2060 assuming constant C inputs on the basis of RothC and two different initialization methods. They estimated a decrease of SOC stocks by 2 to 6% for different climate change scenarios, but speculated that future summer droughts could reduce C inputs in grasslands. In a study in Australia, a SOC decrease of 10 to 11% was predicted under the A2 climate scenario for the period 2008 to 2100 for 12 grassland sites with a constant C input using different initialization methods of RothC^26^. For cropland and grassland soils in Louisiana, a RothC simulation under different climate scenarios and unchanged C input revealed SOC declines of 11 to 18% and 12 to 17%, respectively^24^. An estimation of SOC changes of agricultural soils of Italy within the 21^st^ century using legacy soil data indicated SOC decreases of 3.6 to 11.5% for different climate scenarios and unchanged C inputs^23^. A European-wide study on the basis of legacy data and different climate scenarios estimated SOC declines between 1980 and 2080 of 10 to 14% for croplands and 6 to 10% for grasslands without C input changes^19^. However, these authors also incorporated a simulation of NPP changes in order to estimate C input changes and found a lower decrease of croplands SOC stocks of 3 to 4% and even a slight increase for grassland due to a strong projected increase of NPP.

Similar studies that assumed increased C inputs due to increased NPP generally found SOC gains. For agricultural soils of northeastern Spain, a mean increase of SOC stocks of 6.3% was estimated for different climate projections and agricultural systems (with a wide range of SOC changes of −12.3 to 32.8%) for the period 2007 to 2087 under the assumption of large C input increases of up to 44%^22^. Lugato *et al*.^21^ also estimated a slight SOC gain of 2% for European agricultural soils in the 21^st^ century. In this study, higher soil respiration under climate change was counterbalanced by an assumed 22% higher crop productivity. In SOC projection studies including all land uses on the global or the European scale, both increases and decreases of SOC stocks were projected for coming decades, but it was generally assumed that NPP increases and associated C input gains would offset or even outperform SOC losses induced by global warming, also in agricultural soils^20^,^25^,^38^,^39^.

From our point of view, assumed C input increases in agricultural soils under climate change is a rather optimistic scenario given rising evidence for negative effects of climate change on crop productivity. In several studies, an increased occurrence of droughts and high temperatures above the optimum of crops and a shortening of the growing season were associated with a reduction of NPP^40^,^41^,^42^,^43^,^44^,^45^,^46^. The exceptional dry and hot year 2003 provided a first hint for such a development, as a dramatic decline of C inputs was determined for 2003 (Fig. 4)^47^. As such years are projected to increase under climate change, the possibility of stagnating or even reduced C inputs should be considered more strongly in SOC projections. Moreover, socioeconomic reasons, e.g. a change of agricultural management as a consequence to changes in the common agricultural policy (CAP) in the EU in the 1990s, were quoted to contribute to observed yield stagnation in Europe in the last 25 years^9^,^42^,^48^. Some authors assumed that future improvement of technology, which was mainly responsible for the obtained yield increases until the 1990s, could lead to a strong increase of crop productivity, but as its drivers are not clear, future technology development is afflicted with high uncertainty^26^,^49^. Despite ongoing technological improvement in the last three decades, particularly related to plant breeding^50^, yields of many crops showed no equivalent increase.

The projected decrease of agricultural SOC stocks is associated with a substantial emission of CO_2_ into the atmosphere. A rough estimation for the entire agriculturally used land in Bavaria revealed emissions of 46 to 164 Mt CO_2_-equivalents for the period 2000 to 2095 (depending on the C input scenario) (Table 3, Fig. 7). On a yearly basis, this would increase the CO_2_ emissions of the agricultural sector of Bavaria by 4 to 12% (based on an estimated CO_2_ emission of approximately 14 Mt in 2012). Given the high projected amount of CO_2_ emitted by agricultural soils, climate-change-induced SOC changes should be included in national C accounting programs. Besides the emission of CO_2_, projected losses of SOM in agricultural soils may have negative effects on several important soil functions, e.g. retention of pollutants, buffering capacity, erosion control, water holding capacity and nutrient supply. Overall, the estimated SOC losses could have detrimental effects on soil fertility and ecosystem resilience, which would in turn aggravate crop productivity and thus residue-derived C input – a positive feedback. This calls for an extension of soil monitoring programs and the implementation of early warning indicators in order to verify projected SOC losses.

In view of these potential risks, there is the need to increase C inputs in order to maintain present SOC stocks. A comparison of relative C inputs (related to the SOC stock) for the reference scenario (current climate conditions) and the climate change scenario indicated a future increase of C inputs of 29% needed to counterbalance increased decomposition of SOM (Fig. 8).

Several options were proposed to enhance C inputs in agricultural soils. Promising agricultural practices comprise increased return of crop residues and incorporation of other organic inputs, improved crop rotation including legumes and catch crops, organic farming with clover prominent in the rotation, increased cultivation of bioenergy and perennial crops, improved pasture and livestock management, agroforestry and conversion of cropland to grassland^51^,^52^,^53^,^54^,^55^,^56^,^57^,^58^,^59^,^60^. Recently, such C sequestration options were proposed to offset global anthropogenic CO_2_ emissions in form of the “4‰ concept” at the climate conference in Paris (COP21). This concept is based on the assumption that a yearly increase of global SOC stocks (first 40 cm of soils except permafrost) of 4‰ by improved management would largely contribute to counterbalance human-induced CO_2_ emissions. However, our results indicate that a distinct increase of C inputs by improved agricultural management would be necessary in future decades only to counterbalance enhanced SOM decomposition. Therefore, the potential contribution of improved management of agricultural soils to decrease the atmospheric rise in CO_2_ should be evaluated more carefully.

Although our results provide a good estimate on the potential trajectories of SOC storage in temperate agricultural soils under the given assumptions of climate change and C inputs, model predictions need independent verification and a calculation of uncertainty. There are different sources of uncertainty that may contribute to the final uncertainty in predictions, for instance, uncertainty in model parameters, and in model structure. Although the RothC model was developed for temperate agroecosystems and has been applied successfully in regions under similar climatic and pedogenic conditions in the past, it is still unknown whether RothC’s default parameters may need to be adapted for the study region. Also, the existence of a completely inert pool has been challenged^61^,^62^, and the potential slow dynamics of this pool may become relevant for long-term predictions.

Particularly relevant sources of uncertainty for these types of predictions are the temperature- and moisture-dependent functions implemented in this model to modify decomposition rates. The rate modification by temperature is identical for each SOM pool in RothC despite indications for varying temperature sensitivity of labile and stable SOM pools. The temperature sensitivity of differently stabilized SOM pools is an element of uncertainty in current SOM turnover models and is thus a highly topical debate with regard to global change^2^,^63^. The feedback between climate change and C losses from soil would be much stronger than suggested by equal temperature sensitivities of all SOM pools – like in our calculations, if stable SOM pools react more sensitively to warming than more labile SOM pools. In our studied soils the stable SOM pools amounted for 66 to 90% of total SOC. According to the Arrhenius equation stable SOM pools are thought to be more temperature sensitive than less stabilized SOM pools^3^,^64^. Results from laboratory incubations and long-term experiments indicate that the more stable SOM pools are indeed more temperature sensitive^65^,^66^,^67^. A higher temperature sensitivity of the stable SOM pools implies that the projected mean temperature increase of 3.3 °C may lead to even stronger and sustained C losses from soils than suggested by our scenarios. Therefore, there is the need to integrate information on temperature sensitivity in soil carbon models.

Further critical issues are related to C input projections. Although our scenarios cover the range of expected C input development based on yield projections, more spatially and temporarily precise C input scenarios are needed. A combination of sophisticated yield models together with an estimation of future changes of C allocation patterns of crops could be a promising approach to derive more precise C input estimates.

## Materials and Methods

### Study area

**Figure 1:**
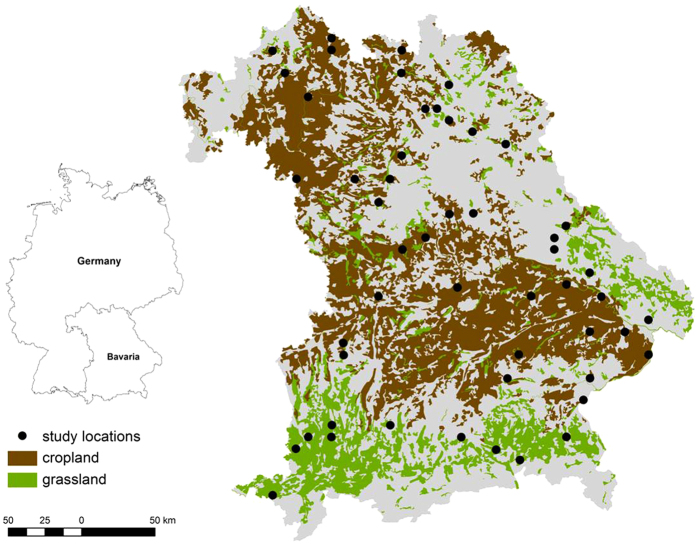
Locations of study sites under cropland and grassland in Bavaria (the map was generated using ESRI ArcMap 9.2, www.esri.com).

**Figure 2:**
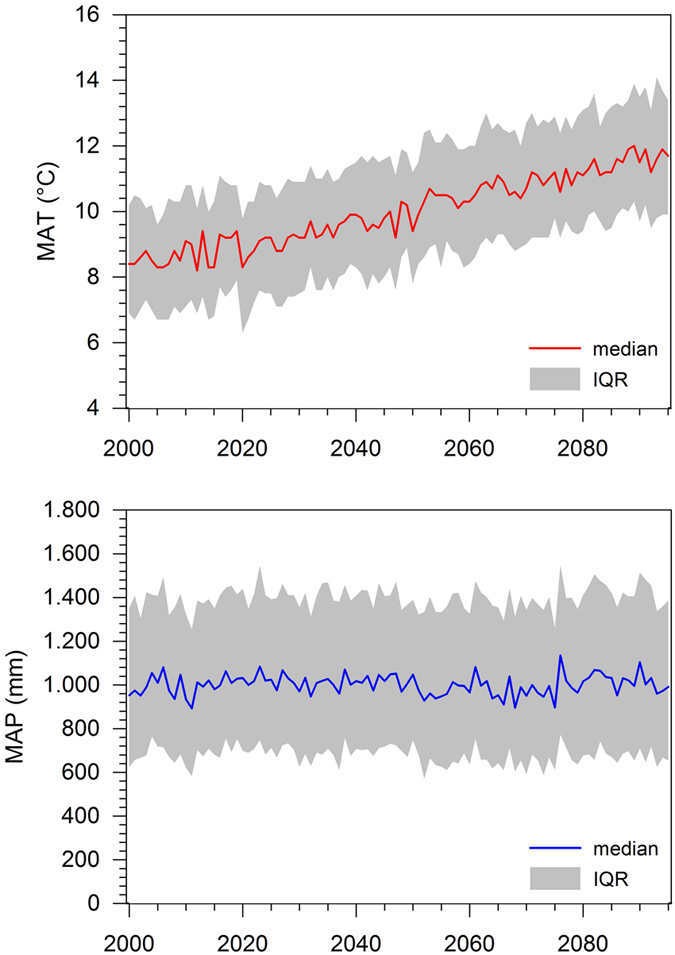
Projected mean annual temperature (MAT) and mean annual precipitation (MAP) for the study sites for the period 2000–2095 under the A1B scenario (median with interquartile range (IQR) derived from 19 climate models).

**Figure 3:**
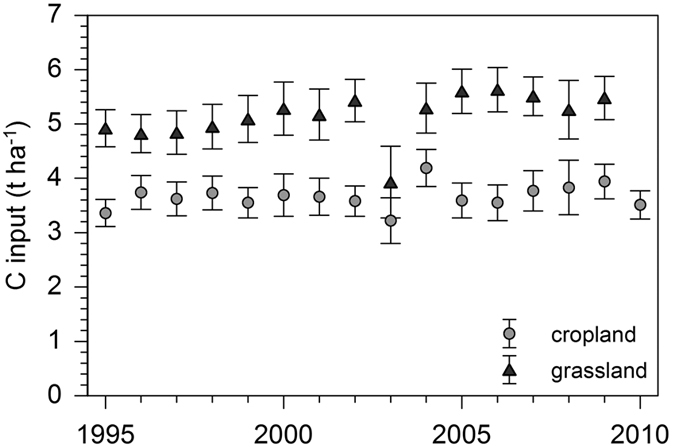
C input in cropland and grassland sites between 1995 and 2010 (mean values with standard deviation).

**Figure 4:**
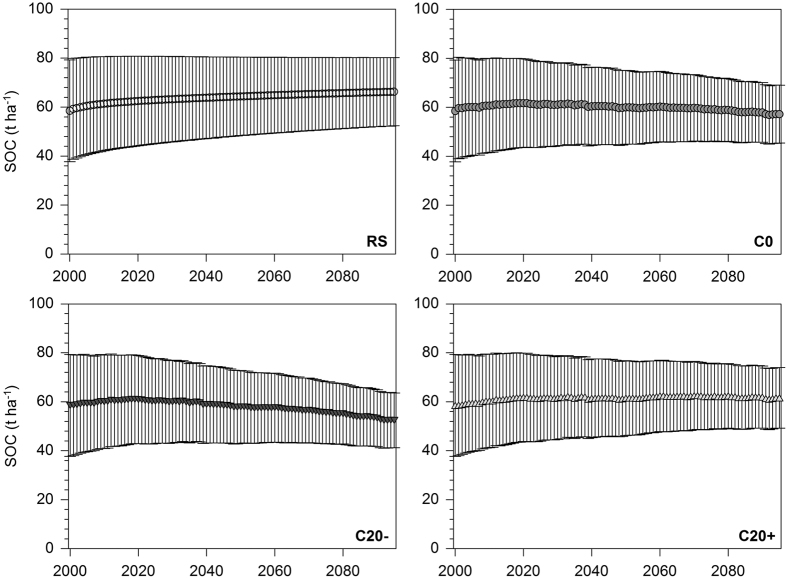
Projected development of SOC stocks of cropland soils in Bavaria between 2000 and 2095 under current climate and land use conditions (reference scenario, RS), climate change and constant C input (C0), climate change and decreased C input by 20% (C20−) and climate change and increased C input by 20% (C20+) (mean values with standard deviation from 21 sites).

**Figure 5:**
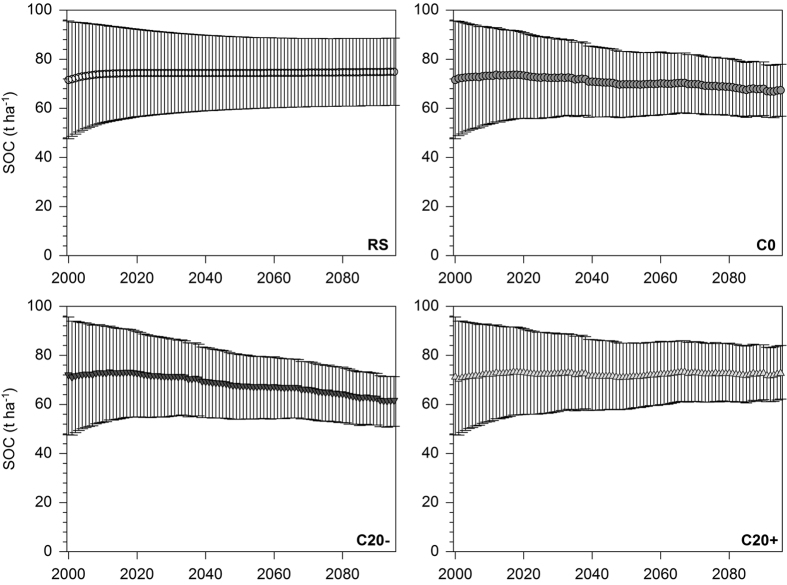
Projected development of SOC stocks of grassland soils in Bavaria between 2000 and 2095 under current climate and land use conditions (reference scenario, RS), climate change and constant C input (C0), climate change and decreased C input by 20% (C20−) and climate change and increased C input by 20% (C20+) (mean values with standard deviation from 30 sites).

**Figure 6:**
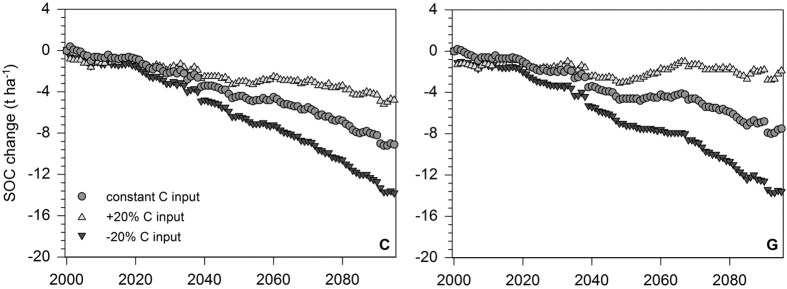
Projected average SOC changes of cropland (C) and grassland (G) sites between 2000 and 2095 under climate change (A1B) and different C input scenarios.

**Figure 7:**
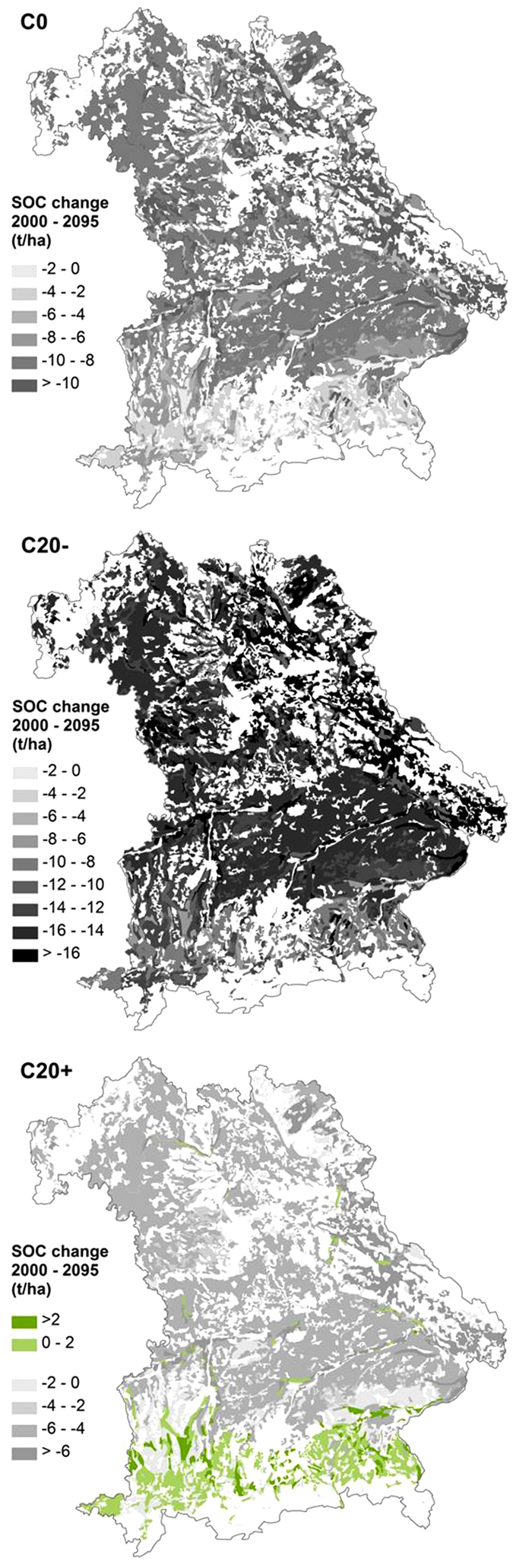
Regional distribution of projected SOC changes in Bavaria between 2000 and 2095 under climate change and constant C input (C0), climate change and decreased C input by 20% (C20−) and climate change and increased C input by 20% (C20+) (the maps were generated using ESRI ArcMap 9.2, www.esri.com/).

**Figure 8:**
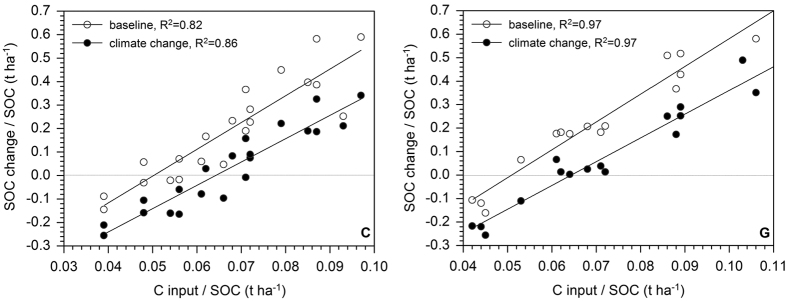
Relative C input in cropland (C) and grassland (G) sites vs. relative projected SOC change under current climate conditions (baseline, 2095) and climate change (A1B, 2095).

The state of Bavaria comprises a total area of 70550 km^2^ and is located in southeast Germany. The northwestern part of Bavaria is dominated by the southern German escarpment landscape that adjoins the low mountain ranges of the Bohemian Massif in the east. Southwards the Molasse basin ascends to the mountain range of the Alps. Elevation ranges between 107 and 2962 m above sea level. Due to its location in central Europe, Bavaria exhibits a sub-oceanic climate that is characterized by a transitional situation between a maritime climate in the northwest and sub-continental influences in the east. Mean annual temperature and precipitation from the escarpment landscape in the northwest to the Alps in the south range between 9° and 4 °C and 550 and 2500 mm, respectively. Around half of the area of Bavaria is under agricultural use, with cropland and grassland accounting for areas of 22843 km^2^ (32% of the total area) and 9897 km^2^ (14% of the total area), respectively (Fig. 1). Dominant soil classes within agricultural land are soils with well-developed B horizons (Cambisols), soils with clay accumulation in the subsoil (Luvisols), soils from limestone weathering with or without loess coverings (Cambisols, Luvisols, Leptosols), clay-rich soils (Cambisols, Vertisols, Stagnosols) and groundwater-affected soils (Gleysols, Fluvisols) according to the German soil system and the equivalent Reference Soil Groups of the WRB system^68^.

Due to the heterogeneous nature in terms of geology, soils and climate, the conditions for agriculture differ considerably within Bavaria^69^. In the south of Bavaria, the region of the Alps and Pre-Alps is characterized by high mean annual precipitation (MAP) of >1000 mm and relatively low mean annual temperatures (MAT) between 5.4 and 7.5 °C. Intensive grassland use for cattle husbandry prevails with up to six swaths per year. The scarce arable land is predominantly cultivated with corn for silage (*Zea mays*). To the north, between the Pre-Alps and the Danube River, the Tertiary Hills Region encompasses a small structured mixed landscape. A more temperate climate with MAT of 7.0–7.7 °C, MAP of 700 to 1000 mm and deep, loamy soils provide good farming conditions. Here, animal husbandry has decreased tremendously over the last 20 years and the conversion of grassland to arable land has been strongest within Bavaria. Corn for biogas production has become a major crop in the rotation together with a mix of other cereals and rapeseed (*Brassica napus*). Within the northern part of the Tertiary Hills Region and further northwest in Bavaria, small, isolated Loess Regions are prominent and form the most productive areas in Bavaria, accompanied by favourable climatic conditions, leading to intensive agricultural production of e.g. sugar beet (*Beta vulgaris*) and winter wheat (*Triticum aestivum*). The East-Bavarian Mid-Range Mountains are characterized again by rather unfavourable climatic and pedological conditions for most high-output cereals. While small areas are used as grassland, potatoes (*Solanum tuberosum*), triticale (×*Triticosecale*), a hybrid of wheat and rye, spring barley (*Hordeum vulgare*) and oats (*Avena sativa*) find good conditions. In the central part of northern Bavaria, the region of Jurassic sediments is characterized by MAT of 7.1–7.2 °C, MAP of around 800 mm and fertile soils. To the northwest, the large northern Bavarian Hill Area exhibits a slightly warmer and drier climate, but sandy, acidic soils and low water availability during the summer months inhibit intensive agricultural production, allowing the cultivation of winter rye (*Secale cereale*) and triticale. Further to the west, the Franconian Lowlands are characterized by lower precipitation (<700 mm), higher MAT (7.5–9.2 °C) and clay-rich, fertile soils, which restrict agriculture to drought-resistant crops like spelt (*Triticum spelta*) and durum wheat (*Triticum durum*) together with winter rye. These grains are also cultivated in the very north-eastern part of Bavaria forming the Franconian Mid-Range Mountains that exhibit a cooler climate (5.2–8.0 °C) and acidic soils but the main use of agricultural land is grassland. Overall, there has been a general trend of intensification of arable land, mainly on more or less productive sites in Bavaria, enhanced through the lifting of the obligatory set-aside policy in 1992, the introduction of a series of renewable energy subsidies since 2003, leading to a huge demand for green maize for biogas production and, last but not least, technological progress in general. On the other hand, there is a visible trend of extensification of marginal sites, where there is a tendency of farmland abandonment, further decrease in livestock numbers and remaining unclear perspectives of farming in the future. Additionally, the steady demand for land for urbanisation in the booming regions of Bavaria is increasing the pressure on agricultural land. However, the share of arable land remained since 20 years and only grassland has decreased substantially, mainly due to compensatory effects for the developments.

### Selection of study sites and soil sampling

Study sites were selected for all major soil units and land uses in Bavaria using a map that combines predominant soil units with main land uses^70^. The map comprises 38 soil units within Bavaria resulting from an intersection of soil type and parent material with macroclimate. These soil units were subdivided to 61 units according to cropland and grassland use on the basis of CORINE Land Cover data. Due to the great effort of the fractionation approach to derive RothC pools, all soil/land use units were excluded which were smaller than 1% of the area of Bavaria. For each of the remaining 18 soil/land use units (8 soil units under cropland and 10 soil units under grassland) representative locations were selected on the basis of available soil material and data from different soil surveys and permanent soil observation sites in Bavaria compiled by the Bavarian Environment Agency (Fig. 1). Although grassland covers only about half the area of cropland in Bavaria, grassland use is distributed over a much wider climatic range and thus more grassland soil units were incorporated in this study. For the majority of soil/land use units, three soil profiles provided representative data. For three of the soil units under cropland (shallow soils from limestone weathering with or without loess coverings; intermediate to deep soils from limestone weathering with or without loess coverings; soils with well developed B horizons from sandstone with initial podzolisation) only two representative soil profiles were available. In total, 51 study sites (21 long-term cropland sites and 30 permanent grassland sites) were selected. The selected soil profiles comprise permanent soil monitoring sites and a grid sampling of 8 × 8 km within Bavaria between 2000 and 2004. For each soil profile, a representative location was selected within a radius of 500 m around the grid node to achieve a homogeneous sampling area in terms of vegetation, relief, soil type and parent material as well as a central position in the particular land use type. Anthropogenic disturbances in the subsoil were excluded in a pre-exploratory survey using a soil auger. Within the topsoil (A horizons), soil material was collected as a composite sample from four sub-locations located with a radius of 6.6 m and four sub-locations with a radius of 3.8 m around the main soil profile in order to cover the small-scale heterogeneity of the soils. At the main profile, soil horizons were additionally sampled using steel cylinders for the determination of bulk density (BD). The content of rock fragments >2 mm was estimated visually in the soil profiles.

### Fractionation of SOC pools and determination of basic soil properties

**Figure 9:**
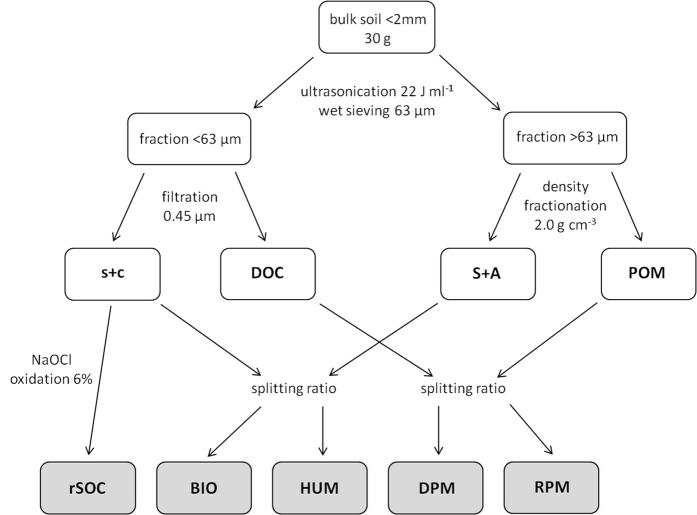
Fractionation scheme of SOC pools according to the method of Zimmermann *et al*.^34^ and Poeplau *et al*.^71^ (s+c = silt- and clay-associated SOM less an inert fraction; DOC = dissolved organic matter; S+A = sand- and aggregate-associated SOM; POM = particulate organic matter; rSOC = inert SOM) and assignment to RothC pools (BIO = microbial biomass; HUM = humified organic matter; DPM = decomposable plant material; RPM = resistant plant material) using splitting ratios.

For each topsoil horizon (A horizons), soil material was fractionated according to the approach of Zimmermann *et al*.^34^ and Poeplau *et al*.^71^ (Fig. 9). Soil material was sieved to 2 mm and 30 g were suspended in 150 ml of deionised water and dispersed using a calibrated ultrasonic probe-type (Bandelin, Berlin, Germany) with an output energy of 22 J ml^−1^. This relatively low energy was applied to disrupt only weakly stabilized soil macroaggregates and to prevent the disruption of mineral-associated SOM^72^. The suspension was wet sieved over a 63 μm sieve until the rinsing water was clear and subsequently filtered through a 0.45 μm membrane filter. The rinsing water was collected and dissolved organic carbon (DOC) was measured using a TOC analyzer 5050A (Shimadzu, Duisburg, Germany). The fractions >0.45 μm and >63 μm were dried at 40 °C and weighted. Afterwards, 50 ml of a sodium polytungstate solution with a density of 2.0 g cm^−3^ 71 was added to the fraction >63 μm and the floating particulate organic matter (POM) was separated after centrifugation at 1000 g for 15 minutes. The remaining fraction >63 μm (sand and stable aggregates, S+A) and the POM fraction were washed with deionised water to remove the sodium polytungstate, dried at 40 °C and weighted. The fraction >0.45 μm (silt and clay, s+c) was homogenized and a subsample of 1 g was oxidized with sodium hypochlorite (NaOCl) to separate a stable SOC fraction. 50 ml of 6% NaOCl, adjusted to pH 8 with concentrated HCl, was added to the subsample and after 18 hours at 25 °C the sample was centrifuged at 1000 g for 15 minutes and washed with deionised water. The oxidation procedure was repeated twice. The residual SOC fraction (rSOC) was dried at 40 °C and weighted. All solid fractions were measured for SOC and N concentration by dry combustion on an EA 3000 analyser (Hekatech, Wegberg, Germany). Soil fractions that contained CaCO_3_ were heated to 500 °C for 4 hours to remove organic carbon, and the concentration of inorganic C of the residual material was determined by dry combustion. The SOC content was calculated by subtracting inorganic C from the total C concentration of the untreated material. For the determination of clay contents, soil samples (<2 mm) were oxidized with H_2_O_2_ to remove organic matter (OM). The remaining material was dispersed with Na_4_P_2_O_7_ and shaken for 16 to 24 hours, followed by wet sieving to isolate sand fractions >63 μm. To determine silt and clay fractions, approximately 3 g of the <63 μm fraction was suspended in deionised water using Na_4_P_2_O_7_ and an ultrasonication for 3 minutes with 75 J mL^−1^ was conducted. Afterwards, the distribution of silt and clay fractions was obtained by measuring the X-ray absorption of the soil-water suspension during sedimentation of the soil particles using a Micromeritics Sedigraph 5100 (Micromeritics, Norcross, GA, USA). For the determination of BD, the mass of the oven-dry soil (105 °C) was divided by the volume of the soil cores.

### Estimation of the C input

In a preliminary study^35^, annual C input values were estimated for most important crops as well as grassland in Bavaria on the county-scale for the period from 1951 to 2010. The C input was calculated on the basis of the C allocation approach proposed by Bolinder *et al*.^37^. This method is based on the allocation of NPP to four plant fractions: the agricultural product (R_P_), aboveground biomass excluding R_P_ (R_S_), belowground biomass excluding RP (R_R_) and extra-root C (R_E_). Due to possible changes of the proportions of the C allocation coefficients R_P_, R_S_, R_R_ and R_E_ in the period between 1951 and 2010 induced by progress in plant breeding, improved crop management and fertilization, C allocation coefficients were estimated both for the beginning (1951–1955) and the end (1995–2010) of the considered period. For the period 1951 to 1955, C allocation coefficients were derived from numerous agricultural field studies and reports that were conducted in the post-war period. The proportions of R_P_ and R_S_ were derived from agricultural statistics for Germany from 1951 to 1955 and from Köhnlein and Vetter^73^. The contribution of R_R_ to NPP was estimated on the basis of several field studies which determined the root biomass of different crop plants gravimetrically by washing of soil monoliths^74^,^75^,^76^,^77^,^78^,^79^,^80^,^81^,^82^. The contribution of R_E_ was estimated to be two-thirds of root biomass according to Bolinder *et al*.^37^. Recent C allocation coefficients for the period 1995–2010 were derived from agricultural statistical data for Germany as well as literature data from 1995 onwards^37^,^83^,^84^,^85^,^86^,^87^,^88^. If no data was provided for R_E_, the contribution of rhizodeposition was estimated as described above. Between 1955 and 1995, allocation coefficients were linearly interpolated for each year according to the linear trend of crop yields within this period.

The total C input as the sum of the C input of all plant fractions except the agricultural product (C_S_+C_R_+C_E_) can be calculated using the relative C allocation coefficients R_P_, R_S_, R_R_ and R_E_ as well as the crop yield:


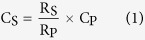



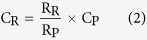



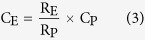


where C_S_ is the C input of aboveground biomass excluding the agricultural product (t ha^−1^ yr^−1^), C_R_ is the C input of belowground biomass (roots) (t ha^−1^ yr^−1^), C_E_ is the C input of rhizodeposition (t ha^−1^ yr^−1^) and C_P_ is the amount of C in the agricultural product (t ha^−1^ yr^−1^). The amount of C in the agricultural product (C_P_) can be calculated using dry-matter yields of respective crops obtained from agricultural statistics assuming that the C content of different plants and plant parts is 0.45 kg kg^−1^ 37. For the estimation of C inputs of the selected study sites, information on the crop rotation, return of harvest residues and the input of organic fertilizers were gained from farmers for each site for a 16-year period (1995–2010). Mean C input values for the initialization of RothC were calculated using C input data derived from 1995 to 1999 before the start of the soil sampling campaign.

### Climate projections

Projections of climate parameters for each study site for the period 2000 to 2095 were derived by using a multi-model ensemble approach on the basis of climate change scenario A1B. The ensemble consists of 19 climate predictions for Germany. They are a combination of different regional climate models (RCM) that are driven by several global climate models (GCM) (Table 4). The multi-model ensemble had been developed in the European framework project ENSEMBLES^89^. As climate models represent reduced natural processes, one model cannot give the full range of information about uncertainties of future climate. To handle these model-based uncertainties the use of ensembles for climate impact studies is widely accepted and recommended^90^. The result of every member of the ensemble is equally probable and represents one possible future but not the only one. The climate data set for the 51 study sites in Bavaria, provided by the German Weather Service (DWD), covers the period from January 2000 to December 2095 and consists of monthly data on air temperature, precipitation and evapotranspiration of the FAO^91^. The latter refers to a short green grass completely shading the ground and with adequate water supply. The spatial resolution of climate data is 25 × 25 km. Every site is represented by a grid cell of 3 × 3 grid points with the study site in the middle. For each grid point median values were calculated for temperature, precipitation and evapotranspiration from the 19 climate projections. To account for spatial insecurity the arithmetic mean of the results of the nine grid points was used for this study.

### Running the RothC model for different carbon input scenarios

To predict the SOC stock development in the topsoil, the RothC 26.3 model was used, which was run in the R environment using the package SoilR^92^. The model simulates the turnover of SOC on the basis of five conceptual pools. Incoming plant material is separated into decomposable plant material (DPM) and resistant plant material (RPM), which are decomposed to humified organic matter (HUM), microbial biomass (BIO) and CO_2_. The HUM and BIO pools undergo further decomposition. Each of these compartments decomposes by first-order kinetics with decay rate constants of 10, 0.3, 0.66 and 0.02 per year for DPM, RPM, BIO and HUM, respectively. Only the inert organic matter pool (IOM) is resistant to decomposition. The decay rate constants are modified by temperature and soil moisture deficit (difference between potential evapotranspiration and precipitation with a maximum deficit defined by the clay content). For model initialization, the measured fractions from the A horizons were summed up and converted to model pools according to the approach of Zimmermann *et al*.^34^ using average RPM/DPM and BIO/HUM splitting ratios for temperate croplands (0.0102 and 0.0272) and grasslands (0.1271 and 0.0259) (Fig. 9). The selected 51 sites were modelled in monthly time steps using the default parameter set of RothC. In order to predict the evolution of SOC stocks from 2000 to 2095, the model was run under unchanged climate conditions (reference scenario, mean climate conditions from the period 1971 to 1999) and a moderate climate change scenario (A1B). The reference scenario served as validation of the estimated C input values. The difference of SOC projections between the reference and the climate change scenario represented the projected SOC change. As a simplification, the measured SOC stock obtained from soil samples taken between 2000 and 2004 was assumed to resemble the stock of the year 2000.

The SOC projections were performed for three different C input scenarios as a realistic range of possible yield development: i) constant C input over time (mean C input from the period 1995 to 1999) (C0), ii) decreasing C input over time (reaching a 20% lower C input in 2095) (C20−) and iii) increasing C input over time (reaching a 20% higher C input in 2095) (C20+). These scenarios were based on present and projected crop yields in this study, which showed a strong correlation with agricultural C inputs so far^35^,^93^. The scenario C0 (constant C input) is based on recent yield stagnation of several crops in Bavaria. Evidence was found that yields of most important crops of Bavaria are stagnating since the 1990s along with stagnating C inputs^9^,^35^. Besides the Status quo of stagnating C inputs, several crop yield modeling approaches projected yield changes within the 21^st^ century. For some study regions in southern Germany, a decline of yields of winter wheat and corn for silage by around 10 to 20% until the mid-century was projected^94^,^95^. On the other hand, an estimation of changes of crop productivity in Europe assumed an increase of yields of major European crops by 15 to 32% from 2020 until 2080 under different climate scenarios (without considering technology development)^49^. In order to cover the full range of expected crop yield development, the scenarios C20− and C20+ were included in this study.

### Statistics

Descriptive statistical methods were used to analyze the soil and climate data sets for maximum and minimum values, mean, median, standard deviation, interquartile range, skewness, kurtosis, variance and coefficient of variation. In order to test the significance of differences of soil properties between the investigated soil units and land uses one-way analyses of variance (ANOVA) combined with post hoc tests (Tukey and Scheffé) and Student’s t-tests were applied. Linear regression analyses were conducted to characterize the trend of C inputs. All statistical calculations were carried out using the software IBM SPSS Statistics 19 (2010, IBM Corp., Armonk).

## Additional Information

**How to cite this article**: Wiesmeier, M. *et al*. Projected loss of soil organic carbon in temperate agricultural soils in the 21^st^ century: effects of climate change and carbon input trends. *Sci. Rep.* **6**, 32525; doi: 10.1038/srep32525 (2016).

## Figures and Tables

**Table 1:** Long-term (1971–1999) mean annual temperature (MAT), mean annual precipitation (MAP), mean annual evapotranspiration (Evap) projected SOC changes (2000–2095) under constant C input (∆SOC C_0_), decreased C input by 20% (∆SOC C_−20%_) and increased C input by 20% (∆SOC C_+20%_) as well as projected changes of MAT, MAP and Evap (2000–2095) of major soil units in Bavaria under cropland (C) and grassland (G) (mean values ± standard deviation).

land use	soil class	MAT (°C)	MAP (mm)	Evap (mm)	∆SOC C_0_ (t ha^−1^)	∆SOC C_−20%_ (t ha^−1^)	∆SOC C_+20%_ (t ha^−1^)	∆MAT (°C)	∆MAP (mm)	∆Evap (mm)
C	G	8.9 ± 0.2	862 ± 63	747 ± 9	−11.8 ± 1.2	−17.1 ± 1.3	−6.9 ± 1.0	3.2 ± 0.1	57 ± 47	118 ± 21
	L2	8.9 ± 0.2	831 ± 20	747 ± 28	−9.4 ± 2.2	−14.0 ± 2.9	−5.2 ± 1.6	3.1 ± 0.1	59 ± 52	105 ± 19
	C1	8.9 ± 0.1	834 ± 55	760 ± 11	−9.3 ± 2.7	−13.9 ± 3.6	−5.2 ± 1.8	3.1 ± 0.1	59 ± 44	123 ± 14
	C3	8.3 ± 0.1	873 ± 79	713 ± 12	−8.5 ± 3.6	−13.4 ± 4.4	−4.1 ± 3.0	3.4 ± 0	82 ± 10	87 ± 10
	C4	8.8 ± 0.1	845 ± 70	754 ± 17	−9.7 ± 1.5	−15.0±1.4	−4.8 ± 1.7	3.2 ± 0	50 ± 8	113 ± 1
	C6	8.8 ± 0.2	840 ± 40	744 ± 8	−8.6 ± 1.7	−13.0 ± 2.6	−4.4 ± 1.0	3.3 ± 0.1	51 ± 11	114 ± 31
	C7	8.3 ± 0.5	864 ± 105	707 ± 47	−4.8 ± 4.6	−9.3 ± 4.1	−0.7 ± 5.0	3.3 ± 0.1	62 ± 5	87 ± 28
	V	8.4 ± 0.2	831 ± 31	710 ± 25	−9.3 ± 2.7	−14.3 ± 3.4	−4.6 ± 2.3	3.3 ± 0	35 ± 33	80 ± 20
G	G	8.0 ± 0.4	1112 ± 260	683 ± 50	−4.4 ± 7.7	−10.6 ± 7.3	1.3 ± 8.1	3.3 ± 0	−8 ± 92	124 ± 29
	L1	7.6 ± 0.2	1327 ± 60	663 ± 31	−1.0 ± 5.3	−7.3 ± 4.5	4.7 ± 6.0	3.4 ± 0	−27 ± 12	143 ± 10
	L2	7.9 ± 0.5	1129 ± 273	686 ± 50	−5.1 ± 7.7	−11.6 ± 7.6	0.8 ± 7.8	3.4 ± 0	2 ± 83	128 ± 33
	C1	8.5 ± 0.9	1019 ± 231	731 ± 48	−7.0 ± 9.1	−13.4 ± 9.1	−1.1 ± 9.2	3.3 ± 0.1	4 ± 41	139 ± 10
	C2	7.9 ± 0.7	1331 ± 267	669 ± 30	−4.0 ± 6.4	−9.8 ± 6.4	1.2 ± 6.4	3.3 ± 0.1	−36 ± 22	142 ± 3
	C3	8.3 ± 0.6	846 ± 92	719 ± 49	−11.4 ± 5.6	−17.5 ± 6.2	−5.7 ± 5.1	3.3 ± 0.1	60 ± 26	94 ± 21
	C5	8.6 ± 0.2	856 ± 36	739 ± 2	−11.7 ± 1.6	−17.5 ± 2.3	−6.3 ± 0.9	3.2 ± 0.1	95 ± 16	105 ± 14
	C6	8.7 ± 0.3	871 ± 54	727 ± 39	−9.3 ± 1.0	−15.2 ± 1.6	−3.8 ± 0.6	3.3 ± 0	28 ± 33	112 ± 35
	C7	8.0 ± 0.2	875 ± 63	698 ± 16	−10.8 ± 2.3	−17.2 ± 2.7	−4.8 ± 2.0	3.4 ± 0	71 ± 12	77 ± 6
	V	8.2 ± 0.3	906 ± 41	708 ± 24	−10.2 ± 0.6	−16.5 ± 0.8	−4.5 ± 0.4	3.4 ± 0	69 ± 10	80 ± 5

G = groundwater-affected soils (Gleysols, Fluvisols); L1 = shallow to intermediate soils with clay accumulation in the subsoil (Luvisols); L2 = intermediate to deep soils with clay accumulation in the subsoil (Luvisols); C1 = soils with well developed B horizons from Tertiary material (Cambisols); C2 = soils with well developed B horizons from morainal material in places with clay accumulation in the subsoil (Cambisols, Luvisols); C3 = shallow soils from limestone weathering with or without loess coverings (Cambisols, Luvisols, Leptosols); C4 = intermediate to deep soils from limestone weathering with or without loess coverings (Cambisols, Luvisols); C5 = soils with well developed B horizons from acidic material with low base saturation (Cambisols); C6 = soils with well developed B horizons from sandstone with low base saturation (Cambisols); C7 = soils with well developed B horizons from sandstone with initial podzolisation (Cambisols, Podzols); V = clay-rich soils (Cambisols, Vertisols, Stagnosols).

**Table 2:** Depth of the A horizon, clay content, C input, OC amount of soil fractions (DOC = dissolved organic matter, SA = sand- and aggregate-associated SOM, POM = particulate organic matter, SC = silt- and clay-associated SOM less an inert fraction, rSOC = inert SOM) of major soil units in Bavaria under cropland (C) and grassland (G) (mean values ± standard deviation).

land use	soil class	sites (n)	A horizon (cm)	clay (%)	C input (t ha^−1^)	DOC (t ha^−1^)	SA (t ha^−1^)	POM (t ha^−1^)	SC (t ha^−1^)	rSOC (t ha^−1^)	SOC t ha^−1^)
C	G	3	32 ± 7	30 ± 15	4.1 ± 0.3	2.5 ± 0.3	12.6 ± 18.7	4.6 ± 2.0	55.5 ± 14.8	5.7 ± 4.5	80.8 ± 2.5
	L2	3	29 ± 5	27 ± 8	3.5 ± 0.5	1.8 ± 0.3	1.0 ± 0.5	2.4 ± 2.0	36.8 ± 9.4	2.6 ± 0.5	44.5 ± 10.9
	C1	3	29 ± 5	21 ± 6	3.5 ± 0.5	1.3 ± 0.3	1.9 ± 0.8	9.9 ± 8.4	29.3 ± 11.5	4.3 ± 1.7	46.8 ± 2.3
	C3	2	26 ± 8	44 ± 13	3.5 ± 0.5	2.2 ± 1.6	2.3 ± 1.3	4.5 ± 3.4	59.1 ± 31.5	3.6 ± 2.0	71.7 ± 39.9
	C4	2	28 ± 4	47 ± 2	3.8 ± 0.2	1.5 ± 0.9	1.9 ± 0.3	3.8 ± 0.6	45.3 ± 4.9	4.9 ± 1.5	57.4 ± 8.1
	C6	3	32 ± 8	22 ± 10	3.5 ± 0.7	1.7 ± 0.9	2.4 ± 0.3	5.6 ± 1.7	42.5 ± 17.7	4.0 ± 1.3	56.3 ± 21.1
	C7	3	27 ± 6	30 ± 16	3.4 ± 0.4	1.3 ± 0	3.6 ± 2.0	4.2 ± 2.0	24.1 ± 4.1	2.8 ± 0.9	36.0 ± 5.0
	V	2	26 ± 4	52 ± 13	3.6 ± 0.5	2.3 ± 1.5	3.6 ± 1.0	5.7 ± 1.9	54.4 ± 20.3	5.1 ± 3.6	71.0 ± 27.3
G	G	3	16 ± 6	33 ± 13	5.0 ± 0.7	1.6 ± 0.3	45.5 ± 33.3	6.4 ± 2.6	23.8 ± 6.9	2.6 ± 0.9	80.0 ± 27.3
	L1	3	25 ± 5	32 ± 10	5.3 ± 0.3	2.1 ± 0.8	24.8 ± 14.0	2.7 ± 1.1	49.1 ± 12.9	3.7 ± 0.3	82.3 ± 6.6
	L2	3	25 ± 6	32 ± 12	5.2 ± 0.3	2.8±1.2	24.3 ± 14.9	5.2 ± 4.1	37.0 ± 7.4	2.3 ± 0.4	71.6 ± 6.5
	C1	3	19 ± 2	23 ± 9	5.1 ± 0.2	2.4 ± 0.5	16.6 ± 4.0	4.1 ± 1.5	37.6 ± 8.0	4.2 ± 1.5	65.0 ± 10.6
	C2	3	32 ± 6	28 ± 13	5.1 ± 0.3	2.7 ± 0.8	17.3 ± 11.9	4.7 ± 1.2	50.6 ± 14.6	6.7 ± 0.7	82.0 ± 25.3
	C3	3	24 ± 6	37 ± 16	4.5 ± 0.5	1.5 ± 0.8	10.6 ± 16.2	4.7 ± 2.6	47.1 ± 22.1	2.3 ± 0.7	66.3 ± 32.0
	C5	3	20 ± 4	19 ± 2	4.5 ± 0.5	1.7 ± 1.1	11.1 ± 2.8	4.2 ± 4.0	30.8 ± 12.9	4.0 ± 1.8	51.8 ± 20.9
	C6	3	23 ± 7	26 ± 6	4.5 ± 0.5	3.4 ± 3.4	9.5 ± 4.6	7.9 ± 5.2	25.2 ± 8.9	4.7 ± 5.2	50.7±19.4
	C7	3	24 ± 9	36 ± 23	4.9 ± 0.1	2.6 ± 1.9	18.8 ± 18.4	6.9 ± 6.1	44.1 ± 11.9	8.3 ± 9.8	80.6 ± 35.8
	V	3	23 ± 8	45 ± 9	4.6 ± 0.3	2.5 ± 1.4	17.1 ± 4.9	4.1 ± 2.9	58.0 ± 30.7	3.6 ± 1.2	85.2 ± 38.5

G = groundwater-affected soils (Gleysols, Fluvisols); L1 = shallow to intermediate soils with clay accumulation in the subsoil (Luvisols); L2 = intermediate to deep soils with clay accumulation in the subsoil (Luvisols); C1 = soils with well developed B horizons from Tertiary material (Cambisols); C2 = soils with well developed B horizons from morainal material in places with clay accumulation in the subsoil (Cambisols, Luvisols); C3 = shallow soils from limestone weathering with or without loess coverings (Cambisols, Luvisols, Leptosols); C4 = intermediate to deep soils from limestone weathering with or without loess coverings (Cambisols, Luvisols); C5 = soils with well developed B horizons from acidic material with low base saturation (Cambisols); C6 = soils with well developed B horizons from sandstone with low base saturation (Cambisols); C7 = soils with well developed B horizons from sandstone with initial podzolisation (Cambisols, Podzols); V = clay-rich soils (Cambisols, Vertisols, Stagnosols).

**Table 3:** Total storage of SOC (2000) and total projected SOC changes in Bavaria (2000–2095) under constant C input (∆SOC C0), decreased C input by 20% (∆SOC C20−) and increased C input by 20% (∆SOC C20+) according to major soil units under cropland (C) and grassland (G).

land use	soil class	SOC (Mt)	∆SOC C0		∆SOC C20−		∆SOC C20+	
			(Mt)	(CO_2_-eq.)	(Mt)	(CO_2_-eq.)	(Mt)	(CO_2_-eq.)
C	G	11.0	−1.6	−5.9	−2.3	−8.5	−0.9	−3.4
	L2	31.0	−6.6	−13.7	−9.8	−20.4	−3.6	−7.6
	C1	18.7	−3.7	−24.0	−5.6	−35.8	−2.1	−13.2
	C3	19.4	−2.3	−8.5	−3.6	−13.3	−1.1	−4.0
	C4	8.4	−1.4	−5.2	−2.2	−8.0	−0.7	−2.6
	C6	9.1	−1.4	−9.6	−2.1	−14.7	−0.7	−4.7
	C7	5.3	−0.7	−5.1	−1.4	−7.7	−0.1	−2.6
	V	20.0	−2.6	−2.6	−4.0	−5.0	−1.3	−0.4
	total	122.9	−20.3	−74.5	−30.9	−113.4	−10.5	−38.5
G	G	5.8	−−0.3	−1.2	−0.8	−2.9	0.1	0.3
	L1	5.5	−0.1	−0.3	−0.5	−1.8	0.3	1.1
	L2	4.6	−0.3	−3.7	−0.7	−7.1	0.1	−0.6
	C1	9.4	−1.0	−2.6	−1.9	−6.3	−0.2	0.8
	C2	14.6	−0.7	−1.2	−1.7	−2.7	0.2	0.2
	C3	3.8	−0.7	−2.4	−1.0	−3.7	−0.3	−1.2
	C5	9.6	−2.2	−3.0	−3.3	−4.8	−1.2	−1.3
	C6	3.7	−0.7	−8.0	−1.1	−11.9	−0.3	−4.3
	C7	6.2	−0.8	−2.5	−1.3	−4.1	−0.4	−1.0
	V	6.7	−0.8	−3.0	−1.3	−4.8	−0.4	−1.4
	total	70.0	−7.6	−27.8	−13.7	−50.2	−2.0	−7.3
total		192.9	−27.9	−102.3	−44.6	−163.6	−12.5	−45.9

G = groundwater-affected soils (Gleysols, Fluvisols); L1 = shallow to intermediate soils with clay accumulation in the subsoil (Luvisols); L2 = intermediate to deep soils with clay accumulation in the subsoil (Luvisols); C1 = soils with well developed B horizons from Tertiary material (Cambisols); C2 = soils with well developed B horizons from morainal material in places with clay accumulation in the subsoil (Cambisols, Luvisols); C3 = shallow soils from limestone weathering with or without loess coverings (Cambisols, Luvisols, Leptosols); C4 = intermediate to deep soils from limestone weathering with or without loess coverings (Cambisols, Luvisols); C5 = soils with well developed B horizons from acidic material with low base saturation (Cambisols); C6 = soils with well developed B horizons from sandstone with low base saturation (Cambisols); C7 = soils with well developed B horizons from sandstone with initial podzolisation (Cambisols, Podzols); V = clay-rich soils (Cambisols, Vertisols, Stagnosols).

**Table 4:** Overview over the ensemble of 19 regional climate projections as a combination of global circulation models (GCM) and regional circulation models (RCM).

	Country/Institution	GCM	RCM
1	Northern Ireland/Community Climate Change Consortium for Ireland	HADCM3Q16	RCA 3.0
2	France/Meteo France	ARPEGE	ALADIN RM5.1
3	Denmark/Danish Meteorological Institute	ARPEGE	HIRMAM 5
4		BCM	
5		ECHAM 5_r3	
6	Switzerland/ETH Zurich (Eidgenössische Technische Hochschule)	HADCM3Q0	CLM 2.4.6
7	Germany/Helmholtz-Zentrum Geesthacht, Centre for Materials and Coastal Research	ECHAM 5_r1	CLM 2.4.11
8		ECHAM 5_r2	
9	England/Hadley Centre for Climate Prediction and Research	HADCM3Q0	HADRM3Q0
10		HADCM3Q3	HADRM3Q3
11		HADCM3Q16	HADRM3Q16
12	Italy/Intern. Centre for Theoretical Physics	ECHAM 5_r3	RegCM 3
13	Netherlands/Royal Netherlands Meteorological Institute	ECHAM 5_r3	RACMO 2.1
14	Germany/Max Planck Institute for Meteorology	ECHAM 5_r1	REMO 2005
15		ECHAM 5_r2	REMO 2009
16		ECHAM 5_r3	REMO 5.7
17	Sweden/Swedish Meteorological and Hydrological Institute, Rossby Centre	BCM	RCA 3.0
18		ECHAM 5_r3	
19		HADCM3Q3	
